# Patient’s Utilization and Perception of the Quality of Curative Care in Community Health Centers of the Fifth Commune of Bsamako

**DOI:** 10.4103/0970-0218.66882

**Published:** 2010-04

**Authors:** Seydou Fomba, Yang Yang, Huan Zhou, Qiaolan Liu, Pr Ma Xiao

**Affiliations:** West China School of Public Health, Health Education Department, School of Public Health, Sichuan University, China

**Keywords:** Bamako/Mali, community health centers, patients, physicians, quality of health care, satisfaction

## Abstract

**Background:**

Community health centers are an important component of the health system in Mali. Despite the adhesion of the populations and the commitment of the authorities, many things must be done to improve the quality of care provided in those structures.

**Objectives:**

The study aimed to know the patients’ utilization and perception of the curative care in the community health centers of Bamako and the physicians’ satisfaction of their work condition and perspective in the community health sector.

**Materials and Methods:**

A cross-sectional study was conducted in nine community health centers of Bamako in 2008. A total of 270 patients were interviewed through a face-to-face interview. Thirteen physicians took a self-administrated questionnaire relating to their material and financial conditions and their plan for the future.

**Results:**

The sample was characterized by the low literacy (32.6%) and socio-economic level (15.9% of steady income).139 patients claimed the nearness as the reason of the choice of the health center whereas only 51 claimed the health staff skill. The women felt more satisfied than men (*P*=0.005) and illiterates felt more satisfied than bachelors and beyond (*P*=0.034). The patients claimed the reduction of waiting time, the improvement of information and the creation of news services. 30.80% of physicians were satisfied from their material and financial conditions, 38.46% were motivated and 76.92% planned to leave their health center.

**Conclusion:**

Although a high level of satisfaction regarding the provided service was observed, user reported some shortage in the quality of care and claimed a widening of CSCom capability.

## Introduction

Patient’s satisfaction has long been considered as an important component when measuring health outcome and quality of care.(1,2) A satisfied patient is more likely to develop a deeper and longer lasting relationship with their medical provider, leading to improve compliance, continuity of care and ultimately better health outcome.(3,4)

Since December 1990, Mali has adopted a health policy named ‘sector-based health’. One of its components was the extension of the healthcare cover for the population and best access to the care; which leaded to the creation of the community health centers. The community health centers are named as CSComs (Centre de Santé Communautaire) and are composed of one dispensary, one maternity and one drug deposit (pharmacy). It has in-charge of delivering the primary health care to the total population across the country.(5) The health staff of those heath centers are composed of physicians (usually in urban area, and rich rural area) and nurses (in the poor rural area). They are characterized by the lack of experience in general according to their number of years of work (usually it is the first job after their graduation) and great mobility (because of ‘low salaries’ in those structures.

In 2007, there were 785 CSComs functional in Mali, and more than 50% of the curative health is provided by them. This proves the importance of those structures in the health system of Mali. In the last few years, critical opinions about CSComs’ health care have been given. In fact, most of the people think that CSComs have bad practitioners; however, the best ones are in the public hospitals and private clinics. In Mali, the community health centers named as CSCom (from French language) are managed by the community via a management committee who are elected by the members of the community health association (ASACO=Association de Santé Communautaire). The government has a role of technical supervision and assistance in it. So, the management committees are often leaded by the people without any knowledge in health area.

Theoretically, the community health centers are no profit structures; but in practice most of the management committees focus more on the profits of the health center rather than on the quality of care, the patient’s satisfaction and the health staff working conditions. This situation has a set back on the health staff stability, the quality of the health care and the kind of customers frequenting those structures. As a result of this, most of those holding position on that health center staff change their working place. By this fact, most of the patients revealed a lack of confidence toward the health staff which is very important in the patient’s satisfaction and frequentation.(4) The clients of those structures are composed in majority by children and adults without good financial capability because the riches prefer to go to big hospitals and private clinics especially in the capital (Bamako).

Since the setting-up of most of those health centers, they have not added any new service to the first one; even some service disappeared, especially in the poor counties. In addition to the curative care, they provide the preventive care, health promotion, epidemiology surveillance and the collection of the heath data. Some studies have been done about patient’s satisfaction but most of them are focused on maternity (pregnancy surveillance, delivery).(6–8) These studies revealed some problems like the deficiency or lack of orientation service, of intimacy and patent’s information about their health status as well as the process of treatment. From all these findings, we decided to do this research in order to have an idea firstly, about the socio-economic composition of the patient’s, their opinion and suggestions about the curative care in those structures and secondly, the physician’s opinion about their material and financial condition as well as their plan for their future career.

## Materials and Methods

The research took place from August to September 2008 in the fifth commune of the district of Bamako. It was a cross-sectional study in 09 well functioning community health center of the fifth commune (ASACOSAB1, ASACOSAB2, ASACOSAB3, ASACOKAL, ASACOTOQUA, ADASCO, ASACODA, ASACOBADJI and ASACOGUA). This commune is one of the biggest of the six communes of Bamako district with a population of around 308,448 in 2008.(9) The population of that commune reflects well the socio-economic and professional group of the population of the district of Bamako (the Capital): rich, middle class and poor (above all peoples coming from counties and who are looking for an employment). In 2007 its average visits per capita/year was 0.66, higher than the mean of all Bamako which was 0.3.(10)

For patient’s interview, an English language questionnaire was used and all the participants were interviewed once. The questionnaire was administered by the same person (the primary researcher) in Bambara language (national language most used in the capital) and for patients who do not speak Bambara language an interpreter was hired to translate the questions in their language. The questionnaire for physician interview was in French language and it was self-administered by physicians.

The questionnaire covers the standard domain used in North American and European surveys by the other authors, Donabedian(1) and EUROPEP questionnaire(11) for assessing patient’s satisfaction. There were a total of 31 questions asked to the patients. The questions about satisfaction concerned: accessibility to service, organisation of care, information and support, medical care, doctor-patient’ relationship and the different service provided cost. The questions concerning these categories of satisfaction were coded from 1 to 5 respectively for excellent, good, fair, bad and very bad. At those conventional criteria, we added questions about patient’s reason for choosing the concerned community health center and their suggestions for the improvement of the quality of health care in that structure. The questions relative to performing or not of an act were coded as 1 if yes and 0 if no. Demographic questions concerning age, sex, level of education and profession were also asked.

Thequestionnaire for physician’s interview had 19 questions treating the items relative to the professional experience, financial and material conditions, relationship with management committee and his future perspectives.

As it was a cross-sectional study and that the population was heterogeneous; we used the systematic sampling method with K=3 (the mean number of patients per CSCom per day is 30 and we had to interview 10 patients per day). The population of that commune reflects well the socio-economic and professional group of the population of the district of Bamako (the Capital): rich, middle class and poor (above all peoples coming from counties and who are looking for an employment).There were 270 respondents that is to say 30 patients interviewed per CSCom. The study was conducted in the waiting room or in an area out of all external influence coming from third person like health staff member or curious onlooker. The interview was realized after that the patient finished all the procedure of care. All patients aged 18 years and above and the children accompanied of person aged 18 years and above, who visited the 09 structures during the inquiry were included. The patients’ consent was obtained before questionnaire administration. The patients who disagreed or who did not answer all the questions were excluded from 270 questionnaires. The interviewer visited the CScom from Monday to Friday. Every third patient registering to be seen by the doctor was invited to participate and in case he/she disagreed the next patient was selected.

The Statistical Package for the Social Sciences (SPSS) version 13.0 was used to analyze the data.(12) And, a comparative statistics was calculated using chi-square analysis for categorical variables and one-way of variance for continuous variables.

## Results

The response rate for participation in this survey was around 95%. The socio-demographic characteristics of study participants are shown in Table 1. So the sample has more female (148) respondents compared to male (122) respondents. The maximum age of respondents was 85 years whereas the minimum was 18 years. The age group of 20-30 years was the most represented whereas the age group of 50-60 years was the least represented, with respectively 31.9 % that is to say (86) and 10.4 % either (28). The higher percentage of respondents were illiterates that is to say 32.6 (88) whereas the lowest percentage were graduates and beyond that is to say 15.2% (41). Regarding the respondents’ profession, the housewives were the most represented with 30.4% (82) whereas the salaried were less represented that is to say 7.8% (21).

**Table 1:** Demographics of the study participants

Variables	n	%
Sex		
Female	148	54.8
Male	122	45.2
Age		
<20	49	18.1
20-30	86	31.9
30-40	49	18.1
40-50	24	8.9
50-60	28	10.4
≥60	34	12.6
Profession		
Salaried	21	7.8
Students	79	29.3
Housewives	82	30.4
Working class	26	9.6
Retired	22	8.1
Others	40	14.8
Education		
Illiterate	88	32.6
Primary	69	25.6
Secondary	72	26.7
Bachelor and beyond	41	15.2

“Others” for profession mean maid, farmer, unemployed and hookers

Table 2 describes the patient’s reasons for selecting the different CSCom and their suggestions for the improvement of the quality of service. The closeness of the structure has been cited 139 times; being in the CSCom before 54 times; the staff skill 51 times; upon recommendation of somebody else 32 times; knowing a member of health staff 26 times; the cheapness of the care 22 times and the humaness of the staff 14 times. Concerning the suggestions, the patients 100 times mentioned that the service should be maintainned in the same way whereas 211 times the patients claimed some changes. The changes claimed were respectivelly 59 times for the reduction of waiting time in the physician’s waiting room, 49 times for the improvement of the reception and orientation system within the CSCom, 35 times for ‘others’ (increasment of the type and quantity of drugs, overseeing of patients who are on a drip etc..), 33 times for the cost of service especially the laboratory tests, 19 times for hiring more care providers and 16 times for creation of new services (X-ray, ultrasound etc).

**Table 2:** Reasons for CSCom choise and suggestions for improving the quality of services

Variables	n
Reasons of choice	
The closeness of CGConi	139
Having been here before	54
The health staff is skill	51
Upon recommendation of somebody	32
Knowing somebody in the CSCom	26
The health care is cheap	22
The health staff is kind	14
Suggestions for improving the service	
No idea	100
To reduce waiting time	59
To improve the reception and orientation	49
To reduce the price of tickets	33
To hire more care providers	19
To create new services	16
Others	35

“Others” means more drugs (quantity and type), overseeing of patients who are on a drip, the respect of order etc. The reduction of the price of ticket concerned above all, the laboratory tests and drip. The total is more than the sample size because of multiple choice questions.

Table 3 describes the patients’ satisfaction according to the different criteria of EUROPEP and the overall satisfaction i.e the patients’ satisfaction about all the process of curative care from the reception to getting out of the CSCom. This table shows that, the physicians provided less information and support to the patients especially about their illness and the continuty of care because only 37.77% (102 out of 270) were satisfied. The patients were satisfied from the doctor-patients relationship with 94.6% (256 out of 270); from the medical care 94.1% (254 out of 270) patients qualified this section good or excellent; from the accessibility 75.19% (203 out of 270) patients were satisfied; from the organization of care 87% (235 out of 270) patients were satisfied and 86.67% of patients judged the overall satisfaction good or excellent.

**Table 3:** Patient’s satisfaction (excellent+good)

Variable	Level of satisfaction					
	Excellent	Good	Fair	Bad	Very bad	Excellent+good %
Doctor-patients relationship	102	154	7	4	3	94.6
Medical care	104	150	10	4	0	94.1
Information and support	64	38	67	74	27	37.77
Accessibility	89	114	35	28	4	75.19
Organization of care	99	136	19	9	7	87.0
Overall satisfaction	77	157	22	7	7	86.67

Satisfied mean, patients who judge the level of satisfaction good or excellent

Table 4 proves that 8 from 13 physicians were payed by the governement whereras 5 from 13 physicians received their salary from the ASACo. Concerning the physicians’ satisfaction, 4 from 13 were satisfied of their material and financial conditions, 5 from 13 felt motivated in their work and 10 from 13 have good relationship with the ASACo. Only 3 from13 planned to stay with the same community health center.

**Table 4:** Physician’s opinion about their working condition and their plan for future

Variables	Freqency (n)	%
Salary provider		
Governement	8	61.54
ASACo	5	38.46
Satisfaction		
Material and financial satisfaction		
Satisfied	4	30.80
Not satisfied	9	69.20
Motivation		
Motivated	5	38.46
Not motivated	8	61.54
Good links with ASACo		
Good relationship	10	76.92
Bad relationship	3	23.08
Plan for career		
Plan to leave the CSCorn	10	76.92
Plan to stay with the CSCom	3	23.08

There was no statistically significant relationship between the age, the profession and the overall satisfaction. In contrast, women felt more satisfied than men (*P*=0.005) and illiterates felt more satisfied than bachelors and beyond (*P*=0.034).

## Discussion

To measure patients’ satisfaction in the health centers and the practitioner’s satisfaction about their working conditions is not a simple task. It can be subject to the loss of interest and even suspicion. In fact, the investigator is considered as a spy by the patients, an evaluator by the health staff and as an outside element who want to interfere in their matter by the ASACo. Nevertheless, evaluation of the quality of care must be both objective and subjective, and the latter is related to the users.

Analysis of the characteristics in the sample has indicated that women were more represented, which can be explained by their availability for accompanying children to the health centers and their prevalence of illness compared to the men.(13) The same was found by Stephen A. Margolis and col(14) with 57% of the women in United Arab Emirates; Binod Kumar Patro and col(15) found 64.4% of the women in New Delhi and Kanta Kadidiatou(8) found out 71% of the women in Segue (Mali). The range 20-30 was the represented because of two factors: the high presence of students who does not have enough resources for going to the private structures and because of some parents who let youngest caring children to the health centers. Illiterates were more represented and can be explained by the weakness of their purchasing power and the high prevalence of illness in that social group.(13)

From this section, we can see that most of the patients are housewives and students. This can be explained by the limited resources of these groups and the fact that housewife usually takes children to visit doctor; salaried in contrast usually go to private clinics for their health care. The improvement of health care quality in the Community Health Center (CHC) may change that situation by solving the problems faced by the patients in those structures as shown in the next section.

Regarding the patient’s reasons for choosing the CSCom, the first three reasons advanced were the near distance of the CSCom, having being in the CSCom before and the skills of the health staff. This is a good signal because all are including the different components of primary health care.(16) That is almost alike to the finding of Otis Ke, Brett J A(17) which indicated that the first 3 barriers for delivering in hospital for Bolivian women were fear or embarrassment 37%, poor quality 22% and distance 21%. The high proportion of no idea in suggestion segment (100 patients) can be explained by the important number of illiterates in the sample. In fact, most of the patients considered themselves as not a health professional and consequently suggested that the questions must be handled by the authorities without asking the populations. Many patients mentioned that the drug shortage as an element to be improved. That is very important, because the drug availability is an important element of the continuity of care above all in developing countries where most people do not have enough resources to purchase medicines from private pharmacies. Mansour A. A(18) found out in Iraq that 50.8% of the patients with diabetes mentioned drug shortage and no drug supply from community health center as the cause for not achieving good glycemia control.

From this section, we can see that the problems faced by the patients of community health center of Bamako are basically poor service quality; long waiting time; lack or inefficiency of reception and orientation system; high price of further analysis and limited number of care givers. So we think that the government should organize a short training course for management committee member’s about service organization; pay the salary for some care givers and set-up the price for all services provided in the C.H.C as it already has done for the prices of medicines.That can make people choosing these health centers for the quality of care provided and not the closeness as we have seen in this research.

The efficacy of medical treatment is enhanced by patient’s satisfaction. In our study, the patients’ satisfaction was high regarding the doctor-patient relationship, medical care, accessibility, organization of care and overall satisfaction; on the other hand, the patients were less satisfied from the information received about their illness, laboratory analysis and treatment. These results correlate with the finding of the other studies.(19–21)

In this section, the main issue is the inefficiency of information and support of physicians to their patients which may be solved by giving them training course about the different segments of the patient’s satisfaction. In fact, this can make them to understand that communication is an important element of medical practice especially in community sector.

Concerning the physician’s opinion about their working conditions and their plan, the satisfaction about the material and financial condition and motivation were low. Even if 10 from 13 thought that they have good relationship with the ASACo, 10 from 13 also planned to leave their health center. Even if the number of respondents is not so important, it is a signal about the problem of the stability of health staff in those health centers. The patients seeing the same physician for long time are more satisfied than patients seeing the first time or second time the same physician.(22,23)

This section proves that, most of the physicians in those strctures are working there while waiting for best job. This situation may be changed by providing some subsides to these structures like the salary of some number of workers (physician, midwife and nurse-in chief).

In our study, the patient’s satisfaction was affected by the gender and education. This is similar to the results of other studies(14,15) but different from the findings of Gadalla in Egypt(21) which mentioned that the patients’ satisfaction was not affected by the gender and education.

## Conclusion

One of the measures of the quality of health care is by assessing client’s satisfaction. This study brought to light that the patients were basically satisfied from physician delivery of care, but wanted some changes according to the current situation. These changes concern the need of new services, hiring more care givers and increasing the health center capability in purschasing medicines. As regard to the physicians, they are not satisfied from their working conditions and most of them plan to leave their health centers. According to the these problems, we suggest that the government subsides the salary for some workers in all the C.H.C; help the management committee to create new services; organize some training course for management committee members about health center’s management; organize traning for physicians about community health care practices; and take measures to standardize the price of services provided in all the C.H.C (according to the law the C.H.C are not for profit making service) and for more involvement of physicians in the daily management of the C.H.C. Those results can be used to direct policy maker decision in the goal to reach the health for all by 2020 as stated by W.H.O (World Health Organization).
